# Supplementary material for: The Combination of MoS2/WO3 and Its Adsorption Properties of Methylene Blue at Low Temperatures
Source: Molecules. 2019 Dec 18;25(1):2. doi: 10.3390/molecules25010002 (PMC6982728; doi:10.3390/molecules25010002)
Supplement: Supplementary file 1 [file molecules-25-00002-s001.pdf]

**Supplementary Materials:** The following are available online. The value of Zeta potential were measured by a ZETA SIZER nano series (Nano-ZS90).

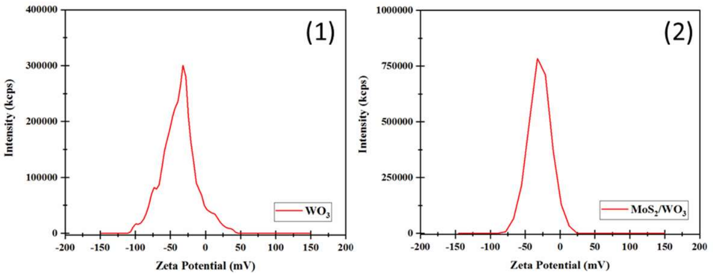


Figure S1. The Zeta potential of $WO_3$ (1) and $MoS_2/WO_3$ (2).
